# Differences in Tolerability of Antifibrotic Agents Between Connective Tissue Disease-Associated and Non-connective Tissue Disease-Associated Interstitial Lung Disease

**DOI:** 10.7759/cureus.78750

**Published:** 2025-02-08

**Authors:** Tomoaki Nakamura, Torahiko Jinta, Atsushi Kitamura, Michiho Tanaka, Clara So, Shosei Ro, Ryosuke Imai, Kohei Okafuji, Yutaka Tomishima, Naoki Nishimura

**Affiliations:** 1 Department of Pulmonary Medicine, Thoracic Center, St. Luke's International Hospital, Tokyo, JPN; 2 Department of Pulmonary Medicine, Thoracic Center, Center Hospital of the National Center for Global Health and Medicine, Tokyo, JPN

**Keywords:** antifibrotic agents, connective tissue disease-associated interstitial lung disease, nintedanib, pirfenidone, tolerability

## Abstract

Background

Although antifibrotic agents (AFAs) are often discontinued due to side effects, their tolerability is crucial given the limited treatment options for interstitial lung disease associated with connective tissue disease (CTD-ILD). The possibility of intolerance due to organ damage caused by CTDs has also been considered; however, few detailed studies are available. We hypothesized that AFAs for CTD-ILD would be poorly tolerated or discontinued prematurely due to organ damage caused by collagen disease. Therefore, we conducted a retrospective investigation to explore this hypothesis.

Methods

We retrospectively reviewed the medical records of ILD patients treated with nintedanib or pirfenidone at St. Luke's International Hospital between December 2008 and November 2022, comparing the CTD-ILD group with the non-CTD-ILD group. Patient background, cumulative discontinuation rate due to adverse events (AEs), duration of prescription until discontinuation, AEs leading to discontinuation, and mortality rate were collected from medical records.

Results

We identified 42 and 129 patients in the CTD-ILD and non-CTD-ILD groups, respectively. The cumulative incidence of discontinuation due to AEs did not significantly differ between the CTD-ILD and non-CTD-ILD groups in the overall population or when restricted to nintedanib and pirfenidone (overall population, p = 0.402; nintedanib group, p = 0.510; pirfenidone group, p = 0.625). The duration of AFA use at discontinuation due to AEs was not significantly different between the overall group (CTD-ILD vs. non-CTD-ILD, median: 77 vs. 116 days, p = 0.496) and the pirfenidone group (CTD-ILD vs. non-CTD-ILD, median: 136 vs. 55 days, p = 0.127). However, for nintedanib, the duration in the CTD-ILD group was significantly shorter than that in the non-CTD-ILD group (median: 23 vs. 218 days, p = 0.016). Gastrointestinal symptoms were the most common reason for discontinuation. Of the seven cases of CTD-ILD patients who used nintedanib and discontinued due to AEs, six were female, four had systemic sclerosis (SSc), and three were using tacrolimus.

Conclusions

The cumulative incidence of discontinuations due to AEs did not significantly differ between the CTD-ILD and non-CTD-ILD groups. However, the duration of nintedanib use was significantly shorter in the CTD-ILD group when the discontinuation was due to AEs. Particularly, when administering nintedanib to patients with SSc and/or those using tacrolimus, measures to prevent AEs should be carefully implemented.

## Introduction

Connective tissue disease-associated interstitial lung disease (CTD-ILD) is a lung disease that can occur in association with CTDs [1]. CTD-ILD is particularly prevalent in diseases such as rheumatoid arthritis and systemic scleroderma, which, despite being different diseases, share similarities in pathophysiology, clinical presentation, and progressive outcome [2]. The primary treatment for CTD-ILD is steroids and other immunosuppressive agents [3]. However, due to the often poor prognosis, antifibrotic agents (AFAs), such as nintedanib and pirfenidone, have been explored as additional treatments [4-7]. In a subgroup analysis of the INBUILD trial, nintedanib slowed the rate of decline in forced vital capacity (FVC) in patients with autoimmune disease-related progressive fibrosing and advanced fibrotic autoimmune disease-related ILD [8]. SENSCIS trial also demonstrated its efficacy in patients with systemic sclerosis (SSc)-ILD [7]. There is multiple evidence supporting the efficacy of pirfenidone. A single-center, prospective, controlled cohort study of patients with CTD-ILD reported a significant improvement in predicted FVC in SSc-ILD or inflammatory myopathy-ILD and in predicted diffusing capacity for carbon monoxide (DLCO) in RA-ILD cases compared with controls [9]. In a double-blind, randomized, placebo-controlled study of SSc-ILDs, FVC stabilized/improved by 94.1% in the pirfenidone group at six months and 76.5% in the placebo group (p = 0.33) [5].

Nevertheless, both pirfenidone and nintedanib have characteristic adverse events (AEs). Pirfenidone is associated more frequently with nausea and rash/photosensitivity, whereas diarrhea and nausea are more frequently reported with nintedanib [10,11]. Real-world data in patients with idiopathic pulmonary fibrosis (IPF) reported discontinuation rates of 20.9% for pirfenidone and 26.3% for nintedanib [12]. However, few studies have investigated whether AFAs are well-tolerated in CTD-ILD. The tolerability of AFAs is an important concern in practice because the range of therapeutic agents is limited. We hypothesized that AFAs for CTD-ILD would be poorly tolerated or discontinued prematurely due to organ damage caused by collagen disease. Therefore, we conducted a retrospective investigation to explore this hypothesis.

## Materials and methods

Patients

This retrospective cohort study included patients with ILD who were treated with either nintedanib or pirfenidone at St. Luke's International Hospital between December 2008 and November 2022. Data collection ceased in April 2023. Exclusion criteria included patients whose data were missing due to AFA being initiated at another hospital, cases where the drug was prescribed for a therapeutic indication but not actually taken by the patient, and cases where the patient was from overseas, and the drug was prescribed at their own expense. Data on patient characteristics, time of death since AFA initiation, and time and reason for discontinuation of AFA were collected. AEs were graded according to the Common Terminology Criteria for Adverse Events (CTCAE), version 5.0 [13].

Patients were divided into CTD-ILD and non-CTD-ILD groups. Diseases in the collagen disease group included rheumatoid arthritis, scleroderma, dermatomyositis/polymyositis, Sjögren's syndrome, systemic lupus erythematosus, antineutrophil cytoplasmic antibody-associated vasculitis, immunoglobulin G4-related diseases, and psoriatic arthritis.

Statistical analysis

Descriptive statistics were used to analyze categorical (frequency and proportion) and continuous (median and range) variables. Categorical variables were compared using Fisher's exact test, and continuous variables were compared using the Mann-Whitney U test. Survival curves post-AFA initiation for the CTD-ILD and non-CTD-ILD groups were assessed using log-rank tests with Kaplan-Meier curves. The actuarial risk of discontinuation due to AEs was estimated using a cumulative incidence function in the presence of death and discontinuation due to non-AEs as competing risks and compared using Gray's test [14]. Additionally, a multivariate analysis was performed using the Fine-Gray regression model, adjusted for age and sex. Patients who survived without documented AFA discontinuation were censored at the last follow-up visit. Statistical analyses were performed using EZR (Saitama Medical Center, Jichi Medical University, Saitama, Japan) [15], a graphical user interface for R Version 4.3.1 (The R Foundation for Statistical Computing, Vienna, Austria). This study was approved by the Institutional Review Board of St. Luke’s International Hospital (approval number 22-R108, approval date December 23, 2022), and the requirement for individual consent was waived.

## Results

**Figure 1:**
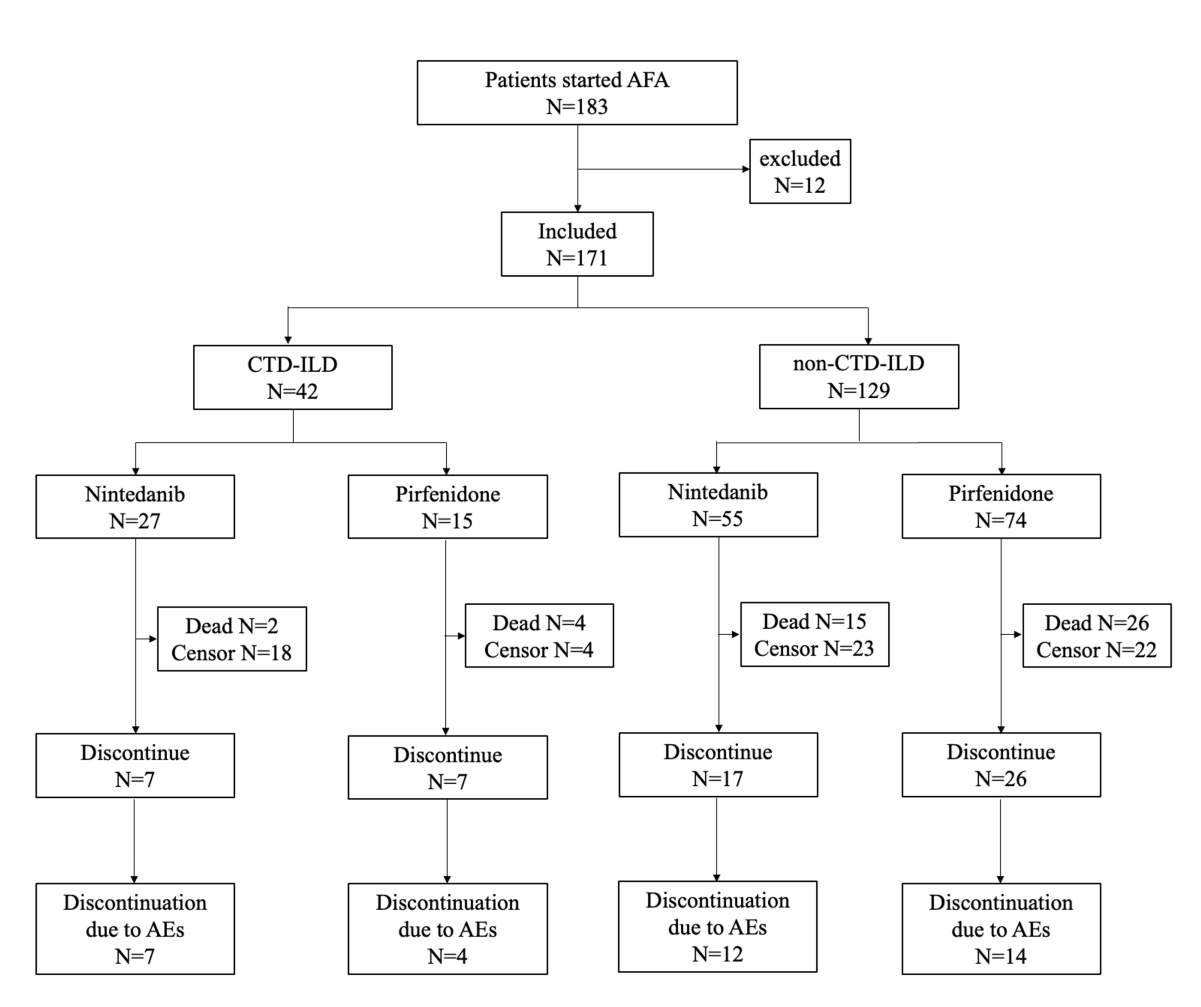
Flowchart of patient selection process AEs, adverse events; AFAs, antifibrotic agents; CTD-ILD, connective tissue disease-associated interstitial lung disease

A total of 183 patients had a history of AFA prescription; however, after applying the exclusion criteria, only 171 patients were finally included. Among these, 42 (25%) were in the CTD-ILD group, whereas 129 (75%) were in the non-CTD-ILD group. Specifically, the number of patients initiated on nintedanib and pirfenidone was 27 (64%) and 15 (36%), respectively, in the CTD-ILD group and 55 (43%) and 74 (57%), respectively, in the non-CTD-ILD group. Among the patients with observed discontinuation events, discontinuations due to AEs were observed in seven patients for nintedanib and four patients for pirfenidone in the CTD-ILD group and 12 and 14 patients for nintedanib and pirfenidone, respectively, in the non-CTD-ILD group (Figure 1).

Table 1 provides an overview of patient background and disease characteristics. The CTD-ILD group had younger patients (median age, 65 years), a higher proportion of women, and a shorter smoking history. Nintedanib was the predominant AFA choice in the CTD-ILD group. However, no significant differences were observed in lung function at the time of AFA initiation.

**Table 1:** Patient and disease characteristics Continuous variables are expressed as median (range), and categorical data are expressed as n (%). Continuous variables were compared using the Mann-Whitney U test, and categorical variables were compared using Fisher's exact test. AE-ILD, acute exacerbations of interstitial lung disease; AFA, antifibrotic agent; ANCA, anti-neutrophil cytoplasmic antibody; CTD-ILD, connective tissue disease-associated interstitial lung disease; DLCO, diffusing capacity for carbon monoxide; FVC, forced vital capacity; HOT, home oxygen therapy

Characteristics	CTD-ILD (n = 42)	Non-CTD-ILD (n = 129)	Test statistic	p-value
Age, years	65 (31-81)	74 (53-90)	W = 1292	<0.001
Male	21 (50)	111 (86)	-	<0.001
Body mass index	21.7 (14.5-34.2)	22.6 (14.7-33.0)	W = 2087	0.576
Pack-years	0.7 (0-120)	35 (0-160)	W = 1632.5	<0.001
AFA				
Nintedanib/pirfenidone	27 (64)/15 (36)	55 (43)/74 (57)	-	0.020
Initiation with a reduced dose of nintedanib	17 (63)	25 (45)	-	0.163
Underlying diseases				
Collagen disease	42 (100)	-	-	-
Scleroderma	23 (55)	-	-	-
Rheumatoid arthritis	7 (17)	-	-	-
Dermatomyositis/polymyositis	7 (17)	-	-	-
ANCA associated vasculitis	5 (12)	-	-	-
Sjögren's syndrome	3 (7)	-	-	-
Systemic lupus erythematosus	3 (7)	-	-	-
IgG4 related disease	2 (5)	-	-	-
Psoriasis	1 (2)	-	-	-
Hepatitis	1 (2)	8 (6)	-	0.456
Liver cirrhosis	1 (2)	2 (2)	-	0.573
Chronic heart failure	16 (38)	52 (40)	-	0.857
Diabetes mellitus	10 (24)	45 (35)	-	0.254
Pneumothorax during lifetime	4 (10)	19 (15)	-	0.603
Thyroid disorder	3 (7)	4 (3)	-	0.365
All types of cancer	12 (29)	48 (37)	-	0.355
Lung cancer	4 (10)	22 (17)	-	0.325
Gastrointestinal cancer	3 (7)	20 (3)	-	0.202
Genitourinary cancer	3 (7)	10 (8)	-	1.000
Head and neck cancer	0 (0)	2 (2)	-	1.000
Breast cancer	0 (0)	1 (1)	-	1.000
Immunosuppressant use	30 (71)	36 (28)	-	<0.001
Steroid	20 (48)	36 (100)		
Tacrolimus	13 (31)	4 (11)		
Mycophenolate mofetil	7 (17)	0 (0)		
Mizoribine	6 (14)	0 (0)		
Abatacept	6 (14)	0 (0)		
Salazosulfapyridine	3 (7)	0 (0)		
Hydroxychlorquine	3 (7)	0 (0)		
Busiramin	1 (2)	0 (0)		
Belimumab	1 (2)	0 (0)		
Cyclosporine	1 (2)	0 (0)		
Sarilumab	1 (2)	0 (0)		
Apremilast	1 (2)	0 (0)		
Cyclophosphamide	0 (0)	2 (6)		
Azathioprine	0 (0)	1 (3)		
Proton pump inhibitor use	18 (43)	53 (41)	-	0.859
Laboratory data at diagnosis				
Albumin, g/dL	4.0 (1.9-4.8)	3.90 (2.0-4.7)	W = 2764.5	0.783
Alanine aminotransferase, U/L	17.5 (7.00-211)	18.0 (8.00-125)	W = 2529	0.519
Aspartate aminotransferase, U/L	21.5 (12.0-69.0)	23.0 (13.0-60.0)	W = 2390.5	0.253
C-reactive protein, mg/dL	0.21 (0.040-7.4)	0.25 (0.040-5.5)	W = 2164	1.000
Krebs von den Lungen-6, U/mL	1097 (289-3176)	930 (245-7567)	W = 2886	0.526
Lactate dehydrogenase, U/L	202 (139-534)	223 (39-570)	W = 2223	0.082
Surfactant protein-D, ng/mL	192.1 (47.30-562.8)	275.3 (51.70-1163)	W = 803	<0.001
Thoracic surgery during lifetime	3 (7)	19 (15)	-	0.290
Lung function test				
%DLCO	57.8 (29.5-99.5)	59.9 (23.5-119.0)	W = 1033.5	0.678
%FVC	79.6 (54.3-122)	80.2 (32.1-125)	W = 1471.5	0.405
%DLCO change before AFA start	-6.1 (-33-8.4)	-7.5 (-46-22)	W = 588.5	0.553
%FVC change before AFA start	-2.9 (-40-12.8)	-7.3 (-55-9.1)	W = 894.5	0.165
Onset of acute exacerbation				
Never	30 (71)	90 (70)	-	0.723
Before using AFA	7 (17)	21 (16)		
In the midst of using AFA	4 (10)	17 (13)		
After using AFA	1 (2)	1 (1)		
HOT during lifetime	10 (24)	55 (43)	-	0.030
Reason for starting AFA				
AE-ILD	3 (7)	20 (15)	-	0.490
Before lung operation	3 (7)	14 (11)		
At diagnosis	2 (5)	6 (5)		
Chronic progression	34 (81)	89 (69)		

**Figure 2:**
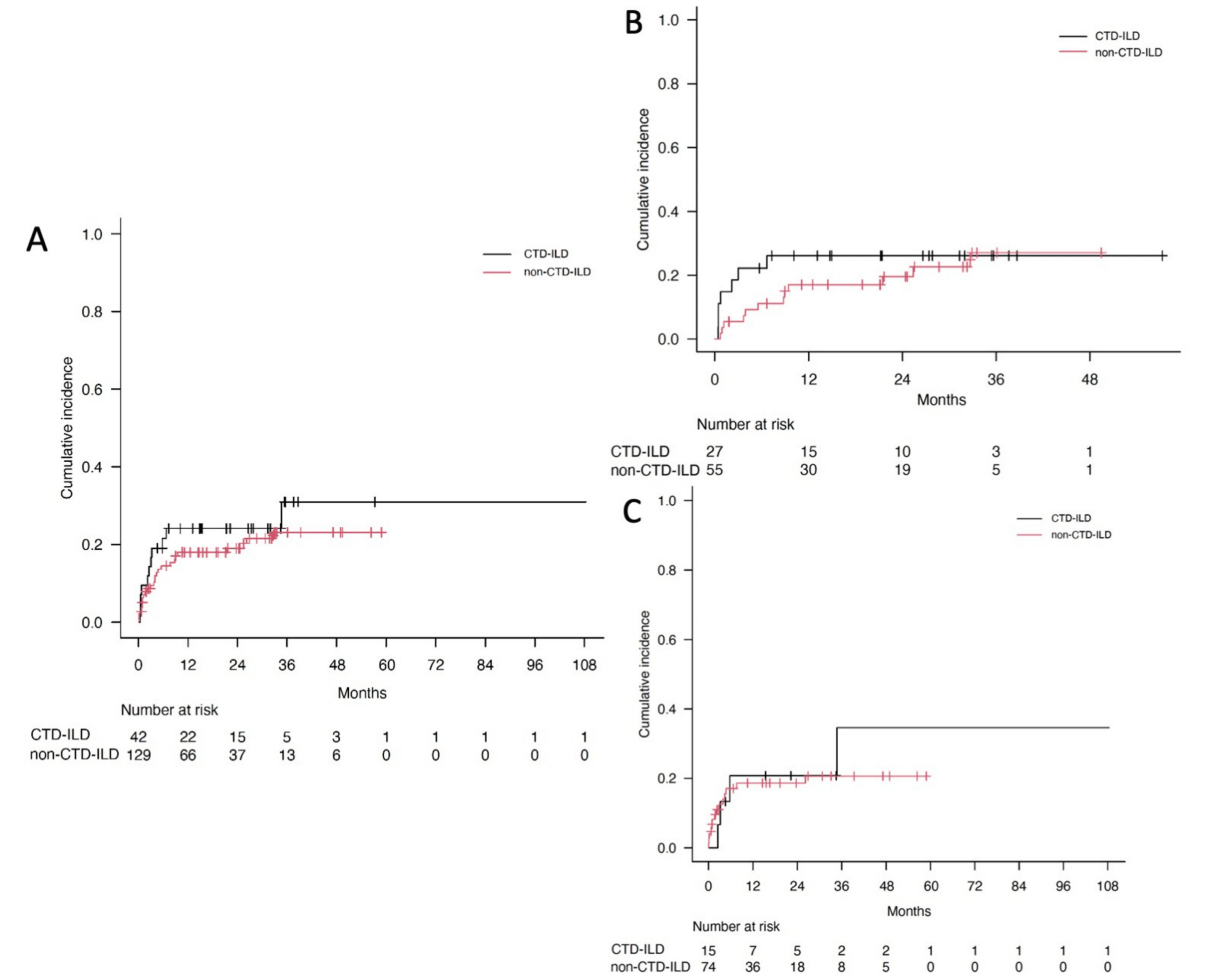
Cumulative incidence of discontinuations of AFAs due to adverse events in the patients in the CTD-ILD and non-CTD-ILD groups (A) Discontinuations due to adverse events, regardless of the type of AFA, were compared between the CTD-ILD and non-CTD-ILD groups. There were no significant differences in incidence (p = 0.402). (B) The study limited the type of AFA to nintedanib (p = 0.510) and (C) pirfenidone (p = 0.625), which showed no significant differences. The actuarial risk of discontinuation due to AEs was estimated using a cumulative incidence function in the presence of death and discontinuation due to non-AEs as competing risks and compared using Gray's test. AEs, adverse events; AFA, antifibrotic agent; CTD-ILD, connective tissue disease-associated interstitial lung disease

The cumulative incidence of discontinuations due to AEs in the CTD-ILD and non-CTD-ILD groups was examined using Gray's test, regardless of the AFA type. No statistically significant difference in incidence was evident (p = 0.402) (Figure 2A). This finding remained consistent when the analysis was stratified by nintedanib or pirfenidone use individually (nintedanib group, p = 0.510; pirfenidone group, p = 0.625) (Figures 2B-2C). Multivariate analysis of the cumulative incidence of discontinuations, adjusted for age and sex, showed that CTD-ILD did not significantly increase the risk of AE-related discontinuation in the overall population or within the nintedanib or pirfenidone subgroups (Table 2).

**Table 2:** Multivariable analysis of the subdistribution hazard ratio for discontinuations due to adverse events in CTD-ILD across populations, using the Fine-Gray regression model CTD-ILD, connective tissue disease-associated interstitial lung disease

CTD-ILD	Subdistribution hazard ratio	95% confidence interval	p-value
All population	1.46	0.682-3.11	0.330
Nintedanib	1.64	0.551-4.85	0.380
Pirfenidone	1.30	0.483-3.49	0.610

The CTD-ILD and non-CTD-ILD groups were compared with respect to the duration of AFA use until discontinuation due to AEs occurred. No significant differences were found between the overall and pirfenidone-treated populations. However, for nintedanib, the duration was significantly shorter in the CTD-ILD group than in the non-CTD-ILD group (Table 3).

**Table 3:** Duration of AFA use in patients who discontinued treatment due to adverse events Variables are expressed as median (range) and are compared using the Mann-Whitney U test. AFA, antifibrotic agent; CTD-ILD, connective tissue disease-associated interstitial lung disease

Duration of AFA use, days	CTD-ILD	Non-CTD-ILD	Test statistic	p-value
All population	77 (13-1056)	116 (1-994)	W = 122	0.496
Nintedanib	23 (13-203)	218 (22-379)	W = 13	0.016
Pirfenidone	136 (77-1056)	55 (1-797)	W = 43	0.127

Gastrointestinal symptoms were the predominant cause of AE-related discontinuations for nintedanib in both the CTD-ILD and non-CTD-ILD groups (Table 4). In contrast, discontinuation of pirfenidone was not only due to gastrointestinal symptoms but also due to photosensitivity and liver injury (Table 5).

**Table 4:** Discontinuation reasons for patients using nintedanib Categorical data are expressed as n (%). AE-ILD, acute exacerbations of interstitial lung disease; CTD-ILD, connective tissue disease-associated interstitial lung disease; GI, gastrointestinal

Discontinuation reason	CTD-ILD		non-CTD-ILD	
	n = 27	CTCAE grading (1/2/3/4/5), n	n = 55	CTCAE grading (1/2/3/4/5), n
Adverse event	7 (26)	0/5/2/0/0	12 (20)	0/6/5/0/0
Diarrhea	5 (71)	0/3/2/0/0	2 (17)	0/1/1/0/0
Anorexia	0 (0)	0/0/0/0/0	5 (42)	0/2/3/0/0
Nausea	1 (14)	0/1/0/0/0	1 (8)	0/1/0/0/0
Vomiting	1 (14)	0/1/0/0/0	0 (0)	0/0/0/0/0
Colitis	0 (0)	0/0/0/0/0	1 (8)	0/1/0/0/0
Colonic perforation	0 (0)	0/0/0/0/0	1 (8)	0/0/1/0/0
Aspartate aminotransferase increased	0 (0)	0/0/0/0/0	1 (8)	0/1/0/0/0
Alanine aminotransferase increased	0 (0)	0/0/0/0/0	1 (8)	0/1/0/0/0
Non-adverse event	0 (0)	-	5 (12)	-
Financial issues	0 (0)	-	2 (40)	-
AE-ILD	0 (0)	-	1 (20)	-
Lung cancer progression	0 (0)	-	1 (20)	-
Other	0 (0)	-	1 (20)	-
Death	2 (7)	-	15 (27)	-

**Table 5:** Discontinuation reasons for patients using pirfenidone Categorical data are expressed as n (%). AE-ILD, acute exacerbations of interstitial lung disease; CTD-ILD, connective tissue disease-associated interstitial lung disease; GI, gastrointestinal; IPF, idiopathic pulmonary fibrosis

Discontinuation reason	CTD-ILD		Non-CTD-ILD	
	n = 15	CTCAE grading (1/2/3/4/5), n	n = 74	CTCAE grading (1/2/3/4/5), n
Adverse event	4 (27)	3/0/1/0/0	14 (19)	0/0/0/0/0
Photosensitivity	2 (50)	2/0/0/0/0	4 (29)	2/2/0/0/0
Anorexia	0 (0)	0/0/0/0/0	5 (29)	0/2/3/0/0
Nausea	1 (25)	1/0/0/0/0	2 (14)	1/1/0/0/0
Vomiting	0 (0)	0/0/0/0/0	1 (7)	0/0/1/0/0
Aspartate aminotransferase increased	0 (0)	0/0/0/0/0	1 (7)	0/1/0/0/0
Alanine aminotransferase increased	1 (25)	0/0/1/0/0	0 (0)	0/0/0/0/0
Pneumothorax	0 (0)	0/0/0/0/0	1 (7)	0/1/0/0/0
Non-adverse event	3 (20)	-	12 (16)	-
Not a diagnosis of IPF	0 (0)	-	3 (25)	-
AE-ILD	0 (0)	-	3 (25)	-
Lung cancer progression	0 (0)	-	1 (13)	-
Other	3 (100)	-	5 (42)	-
Death	4 (27)	-	26 (35)	-

Table 6 presents the details of the seven cases of patients with CTD-ILD who used nintedanib and discontinued due to AEs. Six of the patients were female, four had SSc, and three were using tacrolimus. The starting dose was 200 mg/day for six patients.

**Table 6:** Case series of patients who discontinued nintedanib due to adverse events AFA, antifibrotic agent; BMI, body mass index; CHF, chronic heart failure; CTCAE, Common Terminology Criteria for Adverse Events; CTD, connective tissue disease; DM, dermatomyositis; N/A, not applicable; SSc, systemic sclerosis

Case	Age	Sex	CTD	Immunosuppressant	Underlying diseases	BMI	Dose at the start of AFA, mg/day	Duration of AFA use, days	Discontinuation reason (CTCAE grade)
1	43	Female	DM	Steroid, tacrolimus, abatacept	N/A	22.8	300	13	Diarrhea, (3)
2	54	Female	SSc, DM	Steroid, tacrolimus	Rectal cancer	14.5	200	14	Diarrhea, (2)
3	62	Female	DM, psoriasis	Mizoribine, sarilumab, apremilast	N/A	21.6	200	66	Vomiting, (2)
4	64	Female	DM	Steroid, tacrolimus, mizoribine	Diabetes mellitus	20.5	200	14	Nausea, (2)
5	68	Female	SSc	N/A	N/A	19.6	200	203	Diarrhea, (3)
6	78	Female	SSc, Sjögren ​​​syndrome	N/A	CHF	15.6	200	91	Diarrhea, (2)
7	81	Male	SSc	N/A	Diabetes mellitus, CHF	26.2	200	23	Diarrhea, (2)

**Figure 3:**
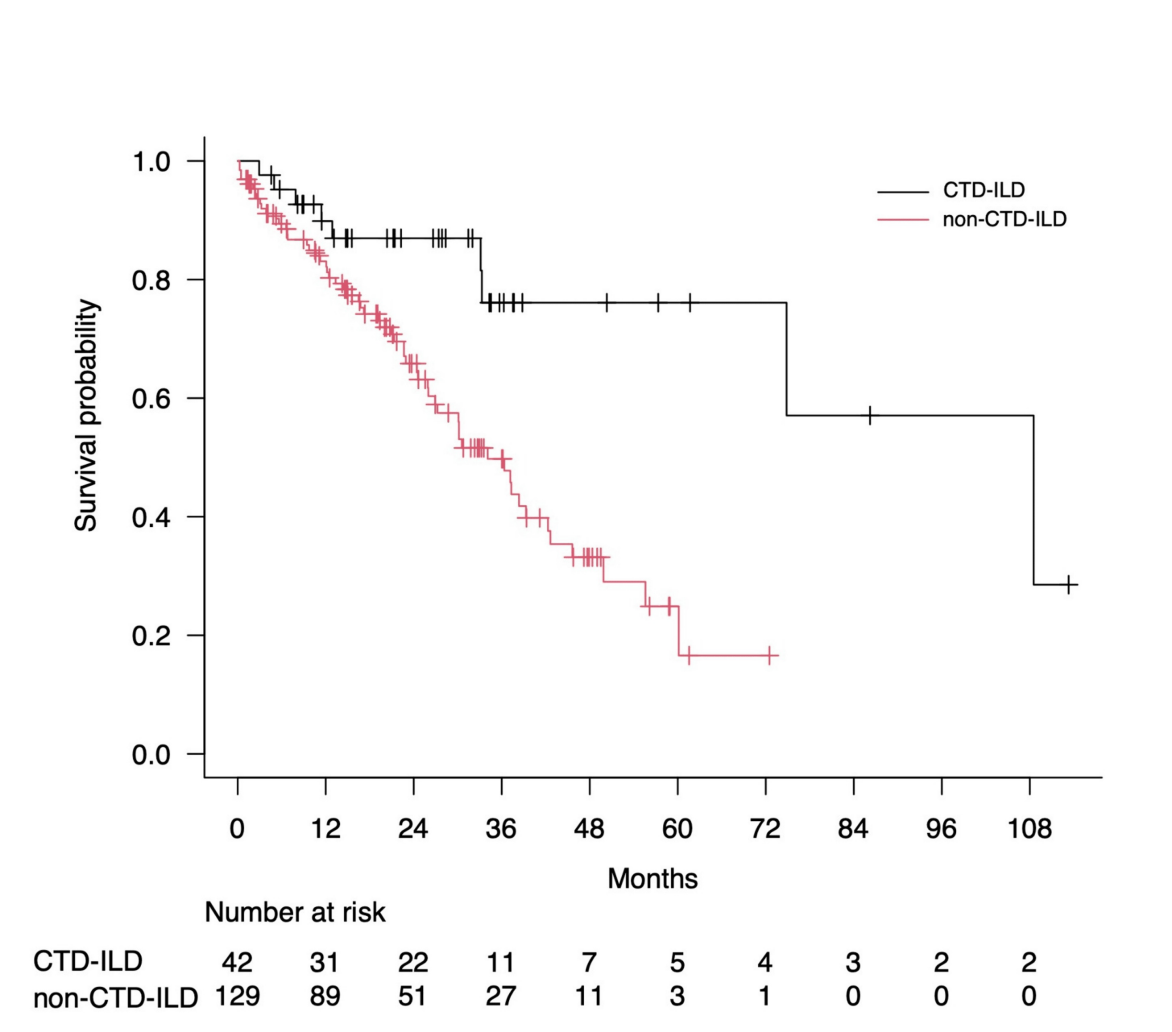
Comparison of the survival curves between the patients in the CTD-ILD and non-CTD-ILD groups Survival curves post-AFA initiation for the CTD-ILD and non-CTD-ILD groups were assessed using log-rank tests with Kaplan-Meier curves. The median survival periods for patients in the CTD-ILD and non-CTD-ILD groups were 108.5 and 34.1 months, respectively (p < 0.001). CTD-ILD, connective tissue disease-associated interstitial lung disease

Comparing the survival curves of patients in the CTD-ILD and non-CTD-ILD groups from the start of AFA use, the prognosis was significantly better in patients with CTD-ILD (p < 0.001); the median survival times were 108.5 and 34.1 months, respectively (Figure 3).

When patients with less than one year of follow-up were excluded, 153 patients remained, and the one-year continuation rate of AFA was examined. The continuation rates of nintedanib were 63% and 60% in the CTD-ILD and non-CTD-ILD groups, respectively (Table 7). For pirfenidone, the continuation rates were 50% and 55%, respectively (Table 8).

**Table 7:** One-year continuation rate of nintedanib Categorical data are expressed as n (%). AE, adverse event

Event	CTD-ILD (n = 24)	Non-CTD-ILD (n = 51)
Continuation	15 (63)	30 (60)
Discontinuation	9 (38)	21 (40)
Due to AE	7 (30)	9 (18)
Due to non-AE	0 (0)	3 (6)
Death	2 (8)	8 (16)

**Table 8:** One-year continuation rate of pirfenidone Categorical data are expressed as n (%). AE, adverse event

Event	CTD-ILD (n = 14)	Non-CTD-ILD (n = 65)
Continuation	7 (50)	36 (55)
Discontinuation	7 (50)	29 (45)
Due to AE	3 (21)	13 (20)
Due to non-AE	2 (14)	6 (9)
Death	2 (14)	10 (15)

## Discussion

In this study, no significant difference was observed in the cumulative incidence of discontinuation due to AEs between the CTD-ILD and non-CTD-ILD groups, regardless of the AFA type. However, the duration of use when discontinuation due to AEs occurred was significantly shorter in the CTD-ILD group than in the non-CTD-ILD group only for nintedanib.

In this study, there was no clear difference in the discontinuation rate of nintedanib due to AEs between the CTD-ILD and non-CTD-ILD groups. In a post-hoc analysis combining data from the INPULSIS, SENSCIS, and INBUILD trials, the discontinuation rate due to AEs in autoimmune disease-related ILDs was 16.5% and 19.6% for other ILDs [16]. A Japanese retrospective study examining the tolerability of nintedanib for SSc-ILDs and idiopathic interstitial pneumonias (IIPs) also found no difference in the permanent discontinuation rates between the two groups (40.7% vs. 32.4%, p = 0.595) [17]. Previous studies have not reported a high discontinuation rate of nintedanib for CTD-ILD, which we consider to be a consistent result.

The duration of nintedanib use until AE-related discontinuation was significantly shorter in the CTD-ILD group than in the non-CTD-ILD group. The reasons for treatment discontinuation were mostly gastrointestinal symptoms. The most common underlying disease in the CTD-ILD group in this study was SSc, which is generally associated with a higher incidence of gastrointestinal symptoms [18]. After steroids, the most commonly used immunosuppressive drug in this study was tacrolimus, which is also known to cause a high frequency of gastrointestinal side effects [19]. Notably, many of the cases shown in Table 5 were either patients with SSc or patients using tacrolimus. Side effects such as nintedanib-induced diarrhea occur relatively early, within three months [20]. Therefore, early discontinuation in the CTD-ILD group may be attributed to the higher number of patients prone to developing gastrointestinal symptoms and that the threshold for discontinuation due to nintedanib administration was easily exceeded. When using nintedanib in patients with these characteristics, side-effect measures should be carefully implemented from the start of administration.

In this study, when discontinued due to AEs, pirfenidone did not have the characteristics seen with nintedanib. The reasons for the discontinuation of pirfenidone varied and included gastrointestinal symptoms, skin rashes, and liver damage. In a study of patients with IPF, gastrointestinal symptoms were less frequent with pirfenidone than with nintedanib, with an increased proportion of the reasons being skin rashes [12]. Therefore, there may have been no obvious differences between the CTD-ILD and non-CTD-ILD groups.

The prognostic differences between CTD-ILDs and IIPs have yielded conflicting results [21-24]. In the present study, the mortality rate was significantly lower in patients with CTD-ILD. Although the time to nintedanib discontinuation tends to be shorter in patients with CTD-ILD, the finding that the CTD-ILD group has a lower mortality rate may seem counterintuitive. However, as shown in Table 7, the one-year discontinuation rate was similar between the CTD-ILD and non-CTD-ILD groups. Additionally, the CTD-ILD group was generally younger, which is considered an advantage for survival. Furthermore, as mentioned above, immunosuppressants are the mainstay of treatment for controlling CTD-ILD disease activity. Therefore, even if AFA is discontinued, it is unlikely to have a significant impact on hard outcomes such as mortality. The mortality difference observed in this study was judged to be a competing risk for the discontinuation of AFA due to AEs. Furthermore, in real-world clinical practice, AFAs are often discontinued due to events other than direct drug-related AEs [25]. We estimated the rate of AFA discontinuation due to AEs using Gray’s test to determine the exact rate. This is one of the strengths of this study.

Our study has some limitations. First, this was a retrospective, single-center study with a small sample size. Further studies with larger sample sizes are required to determine whether these results are consistent. Second, the decision to start or discontinue AFA was left to each attending physician, and there may have been variations in their decisions. Third, this study included cases from 2008 onward, when pirfenidone was first approved globally in Japan. Pirfenidone is approved only for IPF. However, when nintedanib was unavailable worldwide, pirfenidone was prescribed for cases that were not strictly IPF. In this study, pirfenidone was also administered to the CTD-ILD group after being grouped according to the current criteria through chart review.

## Conclusions

In this study, we found no significant difference in the cumulative incidence of AE-related discontinuations between patients with CTD-ILD and those with non-CTD-ILD, regardless of the AFA used. However, the duration of nintedanib treatment before discontinuation due to AEs was notably shorter in the CTD-ILD group. This finding emphasizes the need for heightened vigilance in managing AEs, particularly gastrointestinal symptoms, when initiating nintedanib in patients with SSc or those concurrently using immunosuppressants such as tacrolimus. While no major differences were observed in the tolerability of pirfenidone across groups, its varied AE profile highlights the importance of selecting AFAs based on patient-specific factors.
